# Is Excess Mortality Returning to Pre-Pandemic Levels? A Multi-Model Stochastic Approach for COVID-19: The Spanish Case

**DOI:** 10.3390/epidemiologia7030074

**Published:** 2026-05-26

**Authors:** Julio Ibáñez-Soriano, Francisco G. Morillas-Jurado

**Affiliations:** Departamento d’Economia Aplicada, Universitat de València, Avda Tarongers s/n, 46022 Valencia, Spain

**Keywords:** excess mortality, stochastic mortality models, COVID-19, epidemiology, time-series forecasting, Spain

## Abstract

Introduction: This study quantifies excess mortality in Spain during and after the COVID-19 pandemic and assesses whether mortality levels are returning to pre-pandemic patterns. Methods: Expected mortality was estimated using stochastic forecasting models calibrated on pre-pandemic data (1990–2019) and compared with observed mortality over the period 2020–2023. The analysis relies on a multi-model framework including the Lee–Carter, Cairns–Blake–Dowd, and age–period–cohort models. Results: The results show a substantial excess mortality during the pandemic years, with the proportion of ages exhibiting punctual excess mortality increasing from around 65% before the pandemic to approximately 85% during 2020–2022. Excess mortality declined sharply in 2023, when indicators returned to levels comparable to those observed prior to COVID-19, suggesting a transition toward near-normal mortality. The Lee–Carter model showed superior short-term performance in detecting abrupt mortality deviations, while APC and CBD models captured longer-term structural patterns. Conclusions: These findings highlight the usefulness of multi-model stochastic approaches for monitoring excess mortality and assessing recovery trajectories following major epidemiological shocks.

## 1. Introduction

The COVID-19 pandemic has been an unprecedented global public health challenge. Since its emergence, the virus has affected millions of people worldwide and repeatedly strained the capacity of health systems. Scientific research has been fundamental to understanding the disease, developing vaccines and treatments, and implementing effective prevention and mitigation measures. Numerous studies have addressed the pandemic from different perspectives, including epidemiology [1], immunology [2], mortality shocks [3,4] and forecasting approaches applied to health outcomes [5]. Other related perspectives, such as economic shocks [6,7,8], are beyond the scope of this study.

It is particularly relevant to examine the behaviour of health systems under conditions of elevated epidemiological risk, especially regarding their capacity to respond to sharp increases in morbidity and mortality. During the COVID-19 pandemic, recurrent episodes of healthcare system saturation were observed, characterized by substantial increases in hospital admissions and intensive care unit (ICU) occupancy during periods of high viral transmission [9,10,11]. These dynamics highlighted the role of key clinical risk factors, such as advanced age and pre-existing comorbidities [12,13,14], as well as the seasonal intensification of respiratory infections [15]. Together, these factors contributed to excess mortality and exerted sustained pressure on hospital and critical care services.

In this context, excess mortality has emerged as a key indicator to assess the real impact of the pandemic, beyond officially reported COVID-19 deaths. Excess mortality is typically defined as the difference between the observed number of deaths in a given period and the expected number of deaths for the same period under non-pandemic conditions [16]. This indicator captures both the direct mortality attributable to COVID-19 infection and indirect mortality resulting from delayed diagnoses, reduced access to healthcare, or resource reallocation during periods of system overload.

Measuring excess mortality is important for several reasons. First, it provides a more comprehensive assessment of the overall impact of the pandemic on population health. Second, it enables the evaluation of health system resilience and the effectiveness of public health responses, while offering valuable insights for preparedness and response planning in future epidemic or post-epidemic scenarios.

The aim of this study is to quantify the excess mortality associated with the COVID-19 pandemic in Spain by estimating the difference between observed mortality and the expected mortality in a counterfactual scenario without a pandemic. To achieve this, mortality data from the 30 years preceding the pandemic are used to construct baseline projections, which are then compared with observed mortality during and after the pandemic period.

Projections are generated using stochastic mortality models, including the Lee–Carter model, the Cairns–Blake–Dowd (CBD) model, and age–period–cohort models. The resulting expected mortality trajectories are compared with observed data through a set of quantitative and qualitative indicators.

The data used in this study consist of Spanish mortality records from 1990 to 2019 and mortality data from 2020 to 2024. Data were obtained from the Human Mortality Database (HMD) and the Spanish National Institute of Statistics (INE). Analyses were conducted using R and the StMoMo package.

The results indicate a substantial excess mortality in Spain during the pandemic period. Based on stochastic mortality models calibrated on pre-pandemic data, the proportion of ages exhibiting punctual excess mortality increased from around 65% in the years immediately preceding the pandemic to approximately 85% during 2020–2022, before returning in 2023 to levels comparable to those observed before COVID-19. The magnitude of excess mortality was highest during the first pandemic years, followed by a partial decline in 2021, a renewed increase in 2022, and a marked normalization of mortality patterns in 2023, consistent with a harvesting effect.

## 2. Background and Theoretical Framework

Apart from the own experience of the last years, studies have been performed on the opinions of expert actuaries and demographers on the future behaviour of mortality. In an article about this topic, [17] believe that even without the virus, the steep decline in mortality that occurred during the 20th and early 21st centuries was already stagnating and would deteriorate in the coming years. The factors that explain this are, among others, climate change, population growth, or wars. Not all these factors apply to Spain, but in a globalized society it seems logical that no country should be left out of these events.

In Spain, the “Instituto de Salud Carlos III” (ISCIII) is the international reference entity in Public Health and Biomedical Research. This body prepares the report *Excess of mortality identified by the Daily Mortality Monitoring System (MoMo)* [18], which tries to identify the deviations of daily mortality observed with respect to that expected according to the historical mortality series and allows one to indirectly estimate the impact of any event of importance in public health.

**Figure 1:**
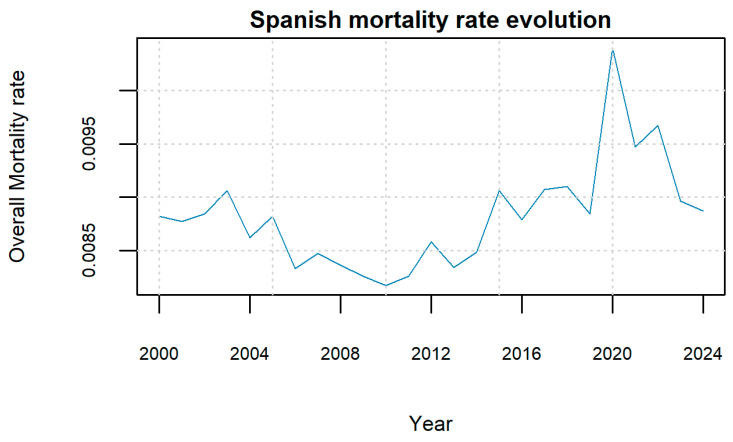
Spanish mortality rate evolution (years 2000–2025). Source: Compiled by authors using data from Human Mortality Database (HMD). The values for 2024 and 2025 are projections.

According to this report, and as shown in Figure 1, the total death rate in Spain has been increasing steadily until 2019, with the slight fluctuations inherent in a process of a stochastic nature. This increase is explained by increase of weight of the population of the most advanced age groups with respect to the total population, groups in which the baby boom generation is already immersed, a phenomenon known as the “inversion of the population pyramid” [19]. This phenomenon is also visible in other European countries [20]. However, in 2020, the year in which the pandemic emerges, we see a sharp increase in mortality that is not explained by the trends up to that point and, therefore, is attributable to the effects of COVID-19.

The “Instituto Carlos III” annually monitors daily excess of mortality (initially caused by climate change, although it now measures excesses due to any cause) through the Daily Mortality Monitoring System (MoMo). The report [18] defines various indicators of excess mortality, which have statistical nature and are measures that attempt to scientifically quantify a characteristic of a sample. Some indicators are qualitative, and others are quantitative. An alert is reported when the number of deaths observed is above the number expected in the analysis period. There are different types of alerts, depending on the degree of difference between expected and actual mortality. An excess of mortality occurs when the observed number of daily deaths exceeds the upper limit of the 99% confidence interval for the expected number of daily deaths. The MoMo defines some quantitative indicators, such as the excess of deaths, which is the difference between the number of observed deaths and the number of expected deaths during the period considered of “excess of mortality”. Another quantitative indicator is the percentage of excess of deaths.

Because the indicators are general and objective, it is possible to have a clear idea of whether an excess of mortality is occurring in a period or not, while at the same time standardizing their analysis to facilitate comparison between periods or regions. We can also have a quantitative sight of the excess in each period.

### 2.1. Data and Methods

In this paper we seek to quantify the effect of COVID-19 on mortality in Spain since, as we have pointed out in Section 2.1, there has been a notable increase in mortality. To do this we project mortality rates for the last 30 years with different stochastic models and see what values would be expected for the period 2020–2025. We use the actual data for these years to compare them, analysing the differences observed using the indicators defined for this purpose.

### 2.2. Stochastic Mortality Models

Stochastic mortality models are used to characterize the evolution of mortality and thus facilitate its study over time. In this work we characterize mortality rates in order to make projections of these rates according to age, period, or cohort. In this section we briefly describe some of the most relevant models and their characteristics. It is usual for these models to consider that the mortality phenomenon is distributed as a random variable with Poisson or Binomial law, by age. An approximation to the underlying theory for these assumptions can be found, for example, in [21].

▪ Lee–Carter model

In 1992, the statisticians Ronald Lee and Lawrence Carter [22] proposed a model to project the mortality rate that differs from other models in that it assumes no cap on life expectancy but allows age-specific mortality to decrease without limit. This method is one of the most widely used and is based on the following expression, where *x* is the age-related subscript, and t is the year-related subscript:

(1) $$\ln\left(m\left(x,t\right)\right)=\alpha_{x}+\beta_{x}*k_{t}+e_{x,t}$$

In Equation (1), $\alpha_{x}$ and $\beta_{x}$ are considered deterministic age-specific parameters estimated from historical mortality data, whereas the stochastic component of the model is represented by the mortality index $k_{t}$. The dynamics of $k_{t}$ are modelled through a univariate stochastic time series process, usually of the ARIMA type. This modelling approach can be used for forecasting purposes. The Lee–Carter model is one of the most widely used in the actuarial world [23,24], and since its publication, different variants have emerged, as well as multiple applications. For example, the Lee–Carter model was adapted to explain the influence of climate on mortality [25]. In [26,27], Hyndman & Ullah propose a functional mortality model as an extension of the Lee–Carter, which allows for smooth functions of age and the amount of noise to become age-specific. Another Lee–Carter adaptation is the Renshaw & Haberman model [28], which is a cohort-based model that incorporates a cohort effect to the Lee-Carter predictor to better capture discontinuities in mortality trends.

▪ Cairns–Blake–Dowd (CBD)

This model [29] differs from the others in that it does not require restrictions on the parameters. In this model the authors assume a binomial distribution for mortality, so the model is expressed with the following equation:

(2) $$logit\left(m_{x,t}\right)=k_{t}^{\left(1\right)}+(x-\overset{-}{x})\cdot k_{t}^{\left(2\right)}+\varepsilon_{x,t}$$

As stated in [30], this model assumes the mortality is linear in the *logit* scale. Consequently, it only works well for older ages, leading to high residuals in early ages and a poor behaviour in general for the residuals. In [31] we find a generalization of the CBD model suggesting that the impact of the cohort effect on a specific cohort decreases over time.

▪ Age–Period–Cohort (APC)

This model (view [32]) considers these three variables (age, period, and cohort) as the determinants of variation in mortality rates in a population. This is the key difference with the other models, although extensions and variations of the Lee–Carter and the CBD have also incorporated these elements. We see the role of each one:

- Age: Collects the variations inherent to biological age and the social processes associated with each age.
- Period: It includes the external effects derived from the historical period in which the population is found. It therefore affects everyone equally. An example of a period effect can be wars, droughts, politics, or environmental changes.
- Cohort: These are variations that affect only a cohort born in a given period. Therefore, the determining variable here is the year of birth. This can be influential in the case of an epidemic affecting only children during a period or a child vaccination program.

Since the cohort variable can be obtained as “period” minus “age”, a linear dependence arises between the variables that gives rise to what is known as the “identification problem”: the difficulty of correctly and independently estimating the effect of each of the three variables. The solution is, for example, to use restrictions on the values of the predictors, as in the Lee–Carter model.

The formulation of this model is as follows:

(3) $$\ln\left(m_{x,t}\right)=\alpha_{x}+k_{t}+\gamma_{t-x}+\varepsilon_{x,t}$$

To avoid the technique bias, we will test the Lee–Carter, the CBD, and the APC models in the data and compare the results to ensure the best model for our data is used.

### 2.3. Goodness of the Models

Some of the most commonly used statistical tools to assess the quality of a model are the information criteria. When models have many possible explanatory variables, as is the case of a mortality model (the variables would be all the ages of each observed year), it is necessary to limit the number of these as too many can add a lot of noise and complexity to the model. At the same time, the model must be reliable and have quality predictors that return a good fit. These information criteria allow us to quantify the balance between predictive capacity and complexity as follows:

IC=complexity−fit

The two main criteria considered are the Akaike information criterion (AIC) [33] and the Bayesian information criterion (BIC) [34]. Both criteria evaluate the goodness of fit of the model while penalizing model complexity through the number of estimated parameters.

Another useful indicator of the goodness of the model is the *deviance*, which is a quality-of-fit statistic for a model that is often used for statistical hypothesis testing. A lower deviance means a better fit to the data.

The AIC, BIC, and *deviance* values were obtained directly from the software implementation. In particular, the R package *StMoMo (version 4.5.1)* [35], provides these criteria for the fitted mortality models considered in this study.

Now, we describe how to determine which models adequately measure excess mortality and can therefore be used in this study. The following process has been structured for this purpose. First, the period for which data is available is divided into subperiods of similar size, [$t_{0}$, $t_{n}$]. Second, for each subperiod, a mortality rate prediction is made for each age with a forecast horizon of h = 5, 10, or 20 years. Third, the predicted mortality rate for each age ($q_{x,t}^{exp}$) is compared with the actual observed values ($q_{x,t}^{obs}$), which were not used in the prediction. To assess the adequacy of the models, the *mean squared error* is considered as the evaluation metric. The sum of these errors, across all considered ages and for all years that make up the validation period (5, 10, or 20 years), constitutes the model’s indicator, which is summarized in Equation (4)

(4) $$e_{c,h}=\sum_{t=t_{n+1}}^{t_{n+h}}\;\sum_{x=0}^{\omega }{\frac{\left(q_{x,n}^{obs}-q_{x,n}^{exp}\right)}{q_{x,n}^{obs}}}^{2}$$

where $e_{c,h}\;$is named the *h-Quadratic mean square error*, and ω is the known *actuarial infinity*.

The models are ordered using the $e_{c,h}$ indicator, and the best model to our purpose is the model with the lowest value in this indicator.

### 2.4. Excess of Mortality Indicators

In Section 2 we show some indicators that the ISCIII uses to measure the impact on Spanish mortality, initially due to climate change. These focus on the daily evolution of mortality by territory, measuring daily confidence intervals or differences between expected deaths and reality.

The objective of our analysis is different, and we cannot use this type of indicators directly. We therefore made an adaptation of the indicators used by the ISCIII in its MoMo report to adapt them to annual mortality broken down by age group as indicated below.

We consider the following indicators to measure the excess of mortality for each age.

Firstly, regarding the *punctual excess mortality (PEM)*, we said that an arbitrary age has a PEM when the mortality rate for this age is higher than the expected value. Then, we consider the *relative excess mortality (REM)*, the percentage by which the actual mortality rate exceeds the expected value. Finally, if the mortality rate for an arbitrary age exceeds the upper limit of the 99% confidence interval of the mortality prediction for that particular age, we said that this age has an *excess of mortality (EM)*.

The mechanism we built to perform the calculations considers the following guidelines:

The main target is to forecast the mortality for the years 2020 to 2025 (estimated data) with the information observed until 2019. Therefore, the forecast horizon is variable, going from 1 to 6 years. To guarantee the reliability of our results, the validation must be made with data forecasted with such horizons.

To perform these validations, the years 2000 to 2019 are forecasted with horizons of 1 to 6 years, which will lead us to compare the results of the year 2020 with the predictions done with a horizon of 1 year, the results of the year 2021 with the predictions done with a horizon of 2 years, etc.

Finally, the results are grouped into 10-year age groups; the mortality indicators of each group are analysed according to the number of indicators by age that have occurred in each group.

### 2.5. About the Data

The Human Mortality Data Base [36] and INE (Instituto Nacional de Estadística, [37]) provide us the mortality rates. In particular, the data used in this study refer to Spain, in the period from 1990 to 2025 (estimated data) and for ages of 0 to 100 years. The databases consist of two matrices, one with the exposed population by age and year of calendar and another with the number of deaths by age and year. Both sets of data are obtained from the Human Mortality Database [36], which in turn is obtained from the respective national statistical institutes of each country. The data for 2022 and 2023 were obtained directly from the INE [37,38,39] since they were not yet included in the HMD. The data for 2024 and 2025 is also from the INE, but it is an estimation since official data is not available yet. A theoretical approximation for the estimations can be found in [2]. Additional data consulted for robustness assessment are described in [40].

### 2.6. Software and Packages

For data analysis we use R-Studio [41] (Version 1.2.5001 and 2025.05.1) and, in particular, the StMoMo package [35], which includes tools for implementing generalized stochastic mortality models such as those we have seen in previous chapters (Lee–Carter, APC, and CBD). It includes functions for fitting mortality models, analysing goodness of fit, and performing mortality predictions and simulations. The demography package [26] has also been used for downloading HMD data. The data and the script developed for the process, the validation step, and the estimation of the h-Quadratic mean square error and to determine the best model are available in [42].

## 3. Results

In this section we derive the main results of the investigation. We apply the models described before to mortality data from Spain in the period prior to the pandemic. We describe the mortality situation and analyse the mortality excesses of reality with respect to the predictions of the models using the indicators defined, which help us to characterize the excess of mortality derived from the COVID-19 pandemic.

### 3.1. Descriptive Analysis

Mortality in Spain is shown for the years of the pandemic, that is, 2019 to 2023. We can observe the logarithmic mortality rates before COVID-19 appeared and its evolution through time.

**Figure 2:**
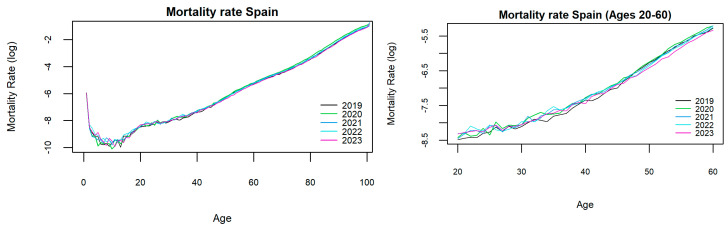
Logarithmic mortality rates of Spain. (**Left** panel): All ages. (**Right** panel): Ages 20 to 60. Source: Elaborated by the authors using their own calculations and data from INE.

The shape of the mortality rate curves we see in the left panel of Figure 2. is the usual for a country like Spain. This shape can be structured as follows: there is a first stage considered (i) *adaptation to the environment*, which represents infant mortality in the first years of life; from birth onwards the mortality rate falls until 4−7 years of age, when it begins to rise. At the end of the first stage, two overlapping stages begin: (ii) *natural mortality*, which lasts until the oldest ages, 80 years and older, and (iii) *the social hump*, also known as the accident hump. This hump is a bulge that occurs at the end of the first stage and can have variations both in amplitude and in the age at which the maximum risk occurs. In this case, the amplitude is from age 17 to 30, with a maximum at age 22. It can be seen that the mortality rate referred to natural longevity is a steadily increasing but stable curve until approaching actuarial infinity.

The evolution of the curves shows high volatility for the lower ages, and in the right panel we can start noticing lower values for the year 2019 with respect to the other years. This goes on until the age of 45, where the year 2023 will continue, until the actuarial infinity, being the year with the lowest mortality, meaning the effects of the pandemic are almost gone. From the age of 45, the year 2020 is the one with highest mortality, showing the big impact in the middle aged and older adult population.

From the age of 50, the curves show very little volatility and do not intersect, each one following its path with different levels of mortality.

From these figures we get a global view of the mortality rates, but the analysis of excess mortality cannot be rigorously performed based on the raw data. For this reason, the articulated process involves applying the previously introduced dynamic mortality models to predict an expected value in a non-pandemic scenario and then comparing them with the observed values and, via the quantitative measures defined to characterize excess mortality, making a more accurate interpretation, as correct as possible. The models selected are Lee–Carter, APC, and CBD.

### 3.2. Model Fitting and Selection for Prediction

To determine the suitability of the models introduced and thus take into consideration one or another model, we use the information criteria introduced in Section 2.3, the AIC and the BIC, as well as the deviance.

Table 1 allows us to ensure that the model that provides the most information for the set of all ages in relation to the available data is the APC. It is this model that has the lowest values in the two information measures considered, AIC and BIC, and in the deviance. The left panel of Table 1 shows the values for the models considering all ages.

It is important to note the difference in magnitude between the models. While the Lee–Carter and APC models have similar magnitudes, the CBD model presents values within the order of 20 times bigger than the other models. According to Diaz Rojo [30], this happens because the CBD model is linear in both the *logit* and the *log* scale; it is usually indicated that this is because this model does not characterize well the adaptation to the environment or the fluctuations of the social hump, so it can be verified that it will work better at older ages. Thus, the panel 2 collects the AIC and BIC values only for ages 70 to 100 years.

Note that although the best models are still APC and Lee–Carter, now the magnitude of the AIC and BIC values has been reduced to only 50% more for CBD, as opposed to the set for all ages, whose value was in the neighbourhood of 2000%.

Once this is resolved, the conclusions provided by the information criteria is that for both age ranges, it is the APC model that has the highest quality, closely followed by the Lee–Carter. The CBD, despite improving when only older ages are considered, continues to be the worst performer according to the AIC and BIC information criteria. In this sense, both Lee–Carter and APC are good candidates to use in our predictions.

Regarding the residuals of the models, the CBD shows clear patterns influenced by the linearity of the model observed previously. Thus, up to 40 years of age, a clear over mortality is detected due to the fact that the model does not correctly reflect the low juvenile mortality. The APC and Lee–Carter show some patterns for some cohorts, but these are not so clear.

The BIC and AIC information criteria do not allow us to differentiate between the Lee–Carter and APC models; they can have similar values for these indicators and, at the same time, not predict well for some ages. To determine whether one or the other model predicts better at certain ages, we use a measure of precision such as the quadratic error. Thus, we use the expression (5) for different years of the period.

In the case of the Lee–Carter model, for a prediction time horizon of 5 years we take data from the period 1990–2010. With these data the model is adjusted, and this adjustment is used to predict the following 5 years, from 2011 to 2015. With the values of the predictions made and the observed values for the same years, the quadratic errors of prediction are obtained:

(5) $$e_{c}\;=\sum_{x=0}^{100}\;\sum_{n=2011}^{2015}{\frac{\left(q_{x,n}^{obs}-q_{x,n}^{exp}\right)}{q_{x,n}^{obs}}}^{2}$$

This would be repeated several times, advancing each time by one year, both in the adjustment interval and in the prediction period, without overlapping (in the second prediction the data used are those of the years 1991–2011, and the projection is made for the years 2012–2016). This makes it possible to quantify the predictive capacity of each model. Applying this process also to the APC and CBD method allows us to select the model to be used in the estimation of excess mortality caused by COVID-19.

As we can see in Table 2, for short- and medium-term projections, Lee–Carter is the best performing model. However, for a long-term projection, the CBD model appears as the best model, although very close to Lee–Carter. Based on these indicators, taking into consideration not only the mean square error but also the values of AIC and BIC and the prediction time horizon for excess mortality estimates (4 years), we determine that Lee–Carter is adequate to perform the analysis.

### 3.3. Excess of Mortality Analysis

In order to accurately determine the excess of mortality that has occurred in each age group, a projection of the models is made for the years 2020 to 2025. These predictions are compared with the actual data on mortality rates, and the indicators defined in Section 3.3 are evaluated. Note that the maximum number of ages with an indicator is 100 for the full year and 10 for the decadal group.

**Figure 3:**
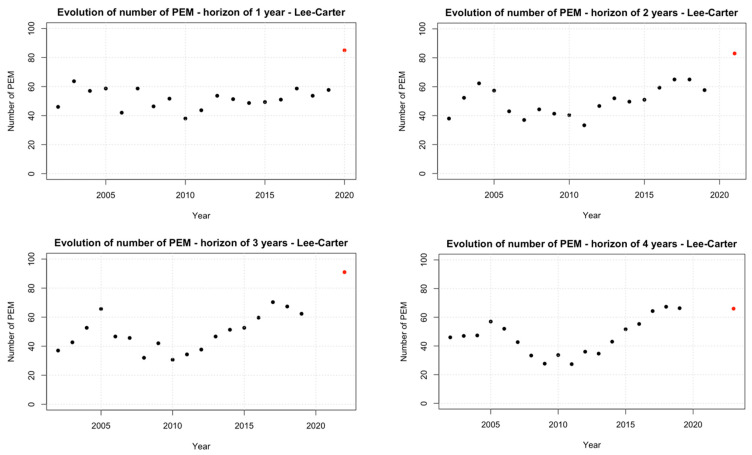
Evolution of number of PEM—Lee–Carter model. (**Top left**): Horizon of 1 year (2020). (**Top right**): Horizon of 2 years (2021). (**Bottom left**): Horizon of 3 years (2022). (**Bottom right**): Horizon of 4 years (2023). Source: Elaborated by the authors using their own calculations and data from INE [24].

First, we encounter the **punctual excess mortality** (PEM), any excess of the actual mortality rate over the one predicted by the projection. In Figure 3. we see the evolution of the number of PEM for every horizon. The years 2015 to 2019 show an increase in the number of indicators, with around 65% of ages having an increase in the mortality with respect to what the models forecasted. In the years 2020 to 2022, this figure reaches 85%. We can observe that in 2023 the PEM returns to the values of the years before the pandemic.

Using the Lee–Carter model to analyse the PEM by 10-year age groups, the punctual excess mortality is shown in Table 3.

When broken down by age groups, the three models considered provide similar values that we interpret as the “harvest effect”. For example, via the Lee–Carter model, in the years 2020 to 2022, for ages 81 to 100 years, there are point excesses of mortality at almost all ages. However, in 2023, this value drops to 2. This could be explained because the first wave of the pandemic was excessively lethal, so that all the people who “probably” would have died during the first two years died along with those who might have survived for one or two more years.

We can also observe a higher excess of mortality in 2022 than in 2021. After a lethal period in 2020, there might have been a general harvest effect, so the mortality fell in 2021, but the new varieties of the virus and the “low” mortality in 2021 made the population vulnerable again, and the mortality rose again in 2022. In 2023 we can observe a big drop, returning to the number of PEM of the years before the pandemic.

**Figure 4:**
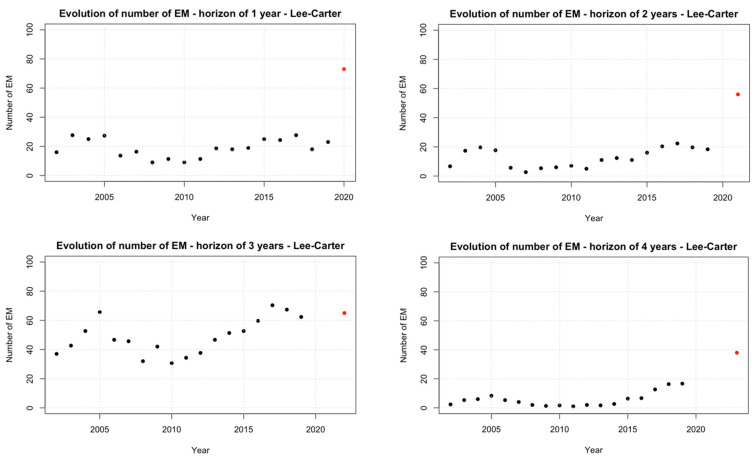
Evolution of number of EM—Lee–Carter model. (**Top left**): Horizon of 1 year (2020). (**Top right**): Horizon of 2 years (2021). (**Bottom left**): Horizon of 3 years (2022). (**Bottom right**): Horizon of 4 years (2023). Source: Elaborated by the authors using their own calculations and data from INE.

Figure 4 shows the **excess mortality** (EM)—defined as the excess of the actual mortality rate over the upper limit of the 99% confidence interval of the prediction—and the values obtained for the Lee–Carter model. We can see the evolution of the number of EM for every horizon, showing that 2020 and 2021 have a big difference with respect to the usual number of EM for the years before the pandemic. Contrary to what happened in the PEM, here 2022 shows little difference with the other years, while 2023, despite having less EM, shows a big difference with the previous years.

If we group the results by decadal age groups, the results are shown in Table 4 for the Lee–Carter model.

Broken down by age and focusing on the Lee–Carter model, in the 21–40 age bracket, EMPs occur at almost all ages. The intermediate ages between young adults and older adults suffer fewer excesses, but from the age of 61 onwards they occur again at all ages in 2020. We can see a strong decrease in the number of EM for these ages that can be explained by the harvest effect, leading to almost no EM at all in 2021 for the segment of 81 to 100. In 2022 we see some EM again, and in 2023, there are no EM for the ages 71 to 100.

Another important indicator of excess mortality is the **relative excesses mortality** (REM) of reality over that expected by the model.

Table 5 shows the REM by age group. The biggest impact occurs in the range of 21 to 40. This can be explained because their mortality in stable conditions is very low; therefore, a small number of deaths in these age groups will cause a shock in their mortality rates. As stated in the other indicators, the harvest effect can be seen here since the REM of the population aged 81 to 100 drops in 2021, and although it rises in 2022, it drops again in 2023. It shows that all weak older adults died in 2020, and the survivors were in better health and could better resist the virus.

### 3.4. Graphical Method

Following the method shown in [43], now we analyse the relative excess mortality from two points of view. One is the REM as we saw it in the indicators, that is, comparing the real mortality rates with the expected ones from the Lee–Carter model. Since the data of every year is very volatile, we used the method of the simple moving average to smooth the function.

**Figure 5:**
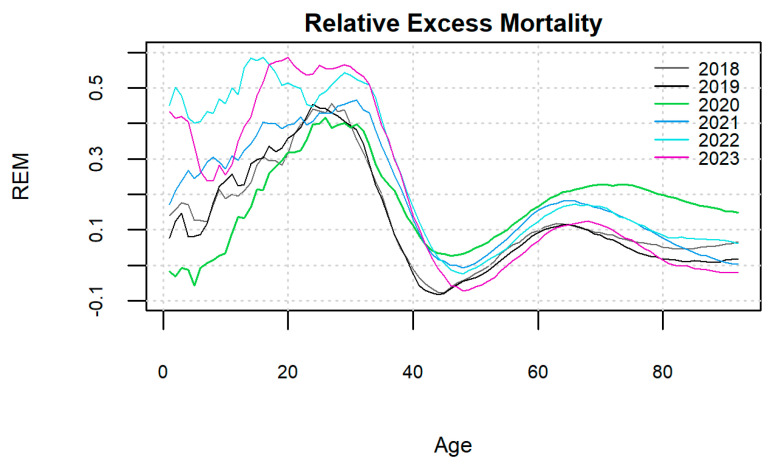
REM by year. Source: Elaborated by the authors using their own calculations and data from INE.

We can see in Figure 5 that even for the years before the pandemic (2018 and 2019), the younger ages have a very high REM. That means the model undervalues the mortality for that range of ages and for those years, leading to an alleged over mortality. We observe a clear high REM in 2020 for the older adults, which then decreases until arriving at normal levels in 2023. The year 2020 is the year with the highest REM from the age of 43 onwards.

For the ages 10 to 35, there is large increase in the REM in 2022 and 2023. This may be explained by three reasons: The first is the low mortality rate in this age range, which leads to big percentual increases in response to a small number of deaths. The second reason is that because people were at home in 2020 and did not travel much in 2020 and 2021, the accident hump was not present in these years, and in 2022 and 2023 it appeared again. The third reason, especially for 2022, is that the vaccines arrived first for the older generations and later for the younger and healthier people, so in 2022, there were two different populations in terms of protection against the virus: one vaccinated and the other non-vaccinated. For every year, there is a big drop in the REM between the ages of 40 and 60.

**Figure 6:**
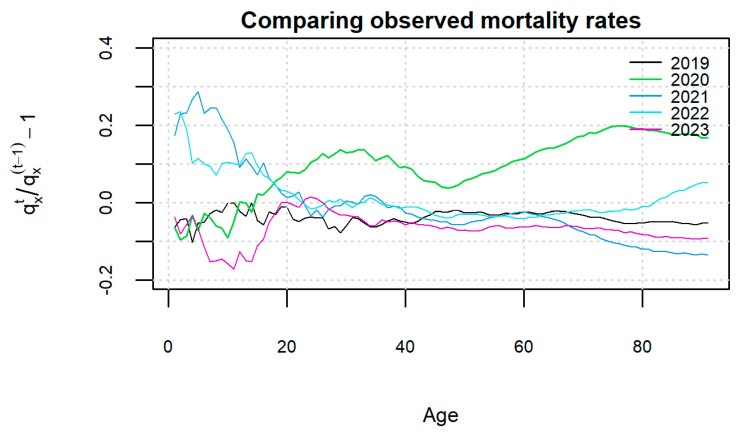
Comparison of excess of mortality using a ratio. Source: Elaborated by the authors using their own calculations and data from INE.

The other point of view to analyse the relative excess of mortality is to compare every year with the precedent year, the “improvement rate”; when this ratio is greater than 1 (0 in logarithmic scale), mortality increases with respect to the previous period, whereas values below 1 (0 in logarithmic scale) indicate a decrease in mortality relative to the previous period. We can see this in Figure 6. 

In this analysis, we focus on the age range from 20 to 100 years. The year 2020 shows a high excess respect 2019. This makes sense, since it is the year when the virus appeared, what caused a big shock in the mortality. For the rest of the years, only 2022 has an excess of mortality, after a 2021 with a large decrease in the mortality for that age range. Looking now at the younger generations, 2021 and 2022 emerge as the years with the highest excess. That means that, after a year of big excess in 2021, the mortality increased again among the population aged 0 to 20. This huge increase caused a big decrease in 2023, when mortality rates came back to normality.

## 4. Discussion

This study provides an in-depth assessment of excess mortality in Spain during and after the COVID-19 pandemic using a multi-model stochastic mortality framework. The results confirm that excess mortality is a robust indicator for capturing both the direct and indirect effects of large-scale epidemiological shocks, particularly in contexts where cause-of-death attribution may be incomplete or heterogeneous over time.

The temporal evolution of excess mortality reflects the dynamic interaction between viral transmission, population vulnerability, and healthcare system capacity. In quantitative terms, the proportion of ages exhibiting punctual excess mortality increased from approximately 65% in the years immediately preceding the pandemic to around 85% during the period 2020–2022, indicating a substantial and widespread mortality shock. By contrast, the sharp decline observed in 2023—when this proportion returned to values comparable to the pre-pandemic period—suggests a transition toward a post-pandemic phase and a normalization of mortality patterns at the population level.

Importantly, excess mortality did not evolve monotonically over time. Following the initial shock in 2020, a partial attenuation was observed in 2021, followed by a renewed increase in 2022 and a marked reduction in 2023. This non-linear trajectory indicates that the mortality impact of COVID-19 extended beyond its acute phase and was shaped by successive epidemiological waves, accumulated delays in healthcare provision, and changes in population-level susceptibility.

The analysis also reveals heterogeneity in the distribution of excess mortality across the population. During the first pandemic years, excess mortality was predominantly concentrated among older individuals, whereas in later stages—particularly in 2022—relative excess mortality increased among younger cohorts. In some adult age groups, relative excess mortality exceeded 50% during 2022, reflecting low baseline mortality and heightened sensitivity to changes in exposure and healthcare access. These shifts are plausibly associated with the restoration of social mobility, the re-emergence of the accident or social hump, differential timing of vaccination coverage, and the delayed effects of postponed medical care.

The pronounced decline in excess mortality among older age groups in subsequent years is consistent with a harvest effect, whereby individuals with higher frailty were disproportionately affected during the earliest pandemic waves. Although this mechanism is clearly visible in aggregated mortality indicators, its precise magnitude cannot be quantified without individual-level longitudinal data.

From a methodological perspective, the multi-model approach confirms that model choice is critical for assessing mortality shocks under conditions of abrupt epidemiological disruption. The superior short-term predictive performance of the Lee–Carter model supports its use for early detection of excess mortality associated with epidemic waves, while the APC and CBD models capture longer-term structural dynamics that are essential for contextualizing post-pandemic mortality patterns. In this respect, the identification of 2023 as the first year of near-normal mortality suggests a re-alignment with expected baseline levels rather than a complete resolution of epidemiological risk.

Taken together, these findings highlight the value of multi-model strategies for robust excess mortality surveillance. Systematic monitoring of deviations from expected mortality trajectories can provide early signals of health system stress and contribute to preparedness and response planning in future epidemic or post-epidemic periods.

## 5. Conclusions

The aim of this study was to quantify excess mortality during the COVID-19 pandemic in Spain, analyse its temporal evolution, and assess whether mortality levels are returning to pre-pandemic patterns. Using a multi-model stochastic mortality framework and a set of quantitative and qualitative indicators, the analysis provides a comprehensive assessment of the magnitude and dynamics of pandemic-related excess mortality.

The findings indicate that COVID-19 mortality in Spain evolved from an acute mortality shock toward a post-pandemic phase characterized by a substantial normalization of mortality patterns. The pronounced decline in excess mortality observed in 2023 supports the interpretation of this year as the first period of near-normal mortality following the pandemic.

The results further confirm that model choice is critical for assessing mortality shocks. The Lee–Carter model showed superior short-term predictive capacity for excess mortality estimation, while the complementary use of APC and CBD models strengthened the robustness of the analysis. Overall, the proposed framework contributes to a clearer understanding of how large-scale epidemiological shocks affect population mortality and provides a useful basis for monitoring recovery trajectories after major public health crises.

### 5.1. Limitations

This study is not without limitations, and these should be taken into account when interpreting the results. Mortality data for the most recent years (2024–2025) are still provisional and may be revised, which introduces uncertainty into the assessment of whether mortality is returning to pre-pandemic levels. The models used assume continuity in pre-pandemic mortality trends, potentially overlooking structural changes in behaviour, healthcare access, and population health conditions that may have arisen during or after the pandemic. In addition, the analysis relies on all-cause mortality and does not allow for distinguishing whether specific causes of death have returned to their pre-pandemic behaviour, a factor that could offer relevant insights into the nature of the observed deviations. The harvest effect, while clearly observable in aggregated data, cannot be precisely quantified without individual-level longitudinal information. Finally, the stochastic mortality models employed do not incorporate socioeconomic or behavioural covariates that may influence differences in the pace at which various demographic groups return to baseline mortality levels.

### 5.2. Improvements

Future research could further explore several aspects arising from this study. A natural extension would be to examine cause-specific mortality to determine whether the patterns identified at the aggregate level also manifest within specific categories of death, providing deeper insight into how different components of mortality have evolved in relation to pre-pandemic levels. Additional work could analyse heterogeneity across regions, socioeconomic groups, and clinical profiles, offering a more granular understanding of the differentiated recovery of mortality patterns. Comparisons with other European countries could help contextualize Spain’s trajectory within wider demographic responses to the pandemic. Methodologically, future studies could develop structural stochastic mortality models that explicitly incorporate pandemic-induced shocks, potentially enhancing the accuracy of long-term projections. Finally, further research could evaluate how the gradual normalization of mortality affects pension systems, life insurance pricing, and healthcare resource planning in the medium and long term. 

## Figures and Tables

**Table 1:** Akaike and Bayesian information criteria of the used models jointly with deviance.

Model	Lee–Carter	CBD	APC	Model	Lee–Carter	CBD	APC
**AIC**	39,273	94,711	35,785	AIC	12,497	18,655	11,758
**BIC**	40,656	94,747	37,300	BIC	12,931	18,944	12,298
**Deviance**	11,822	91,996	8,290	Deviance	2431	8760	1648
Left panel: All ages.				Right panel: Ages 70 to 100			

Source: Compiled by the authors.

**Table 2:** Quadratic error of the models.

Years of Prediction	LC	CBD	APC
**5**	0.2093	1.6655	0.473
**10**	0.6369	2.7291	1.008
**20**	8.5337	7.9965	13.0063

Source: Compiled by the authors. Data for years 2024 and 2025 are projections.

**Table 3:** Number of ages with PEM in Lee–Carter model. (by decadal groups of age).

Age	2020	2021	2022	2023	2024 *	2025 *
**1–10**	8	9	10	10	9	9
**11–20**	4	10	10	7	10	10
**21–30**	10	10	10	10	10	10
**31–40**	10	10	10	10	10	10
**41–50**	4	4	5	5	10	10
**51–60**	8	6	6	2	10	10
**61–70**	10	10	10	10	10	10
**71–80**	10	10	10	10	10	10
**81–90**	10	10	10	2	10	10
**91–100**	10	4	10	0	10	10
**Total**	84	83	91	66	99	99

Source: Compiled by the authors. * Data for years 2024 and 2025 are projections.

**Table 4:** Number of ages which have an EM in the Lee–Carter model.

Age	2020	2021	2022	2023	2024 *	2025 *
**0–10**	3	5	9	9	8	8
**11–20**	3	6	8	3	6	6
**21–30**	10	10	10	10	9	9
**31–40**	9	10	8	9	9	9
**41–50**	1	1	1	0	0	0
**51–60**	6	2	1	0	0	0
**61–70**	10	10	10	7	5	4
**71–80**	10	10	9	0	6	5
**81–90**	10	2	1	0	1	2
**91–100**	10	0	8	0	2	0
**Total**	73	56	65	38	46	43

Source: Compiled by the authors. * Data for years 2024 and 2025 are projections.

**Table 5:** REM by ages in Lee–Carter model.

Age	2020	2021	2022	2023	2024 *	2025 *
**1–10**	5.8%	26.2%	57.4%	56.5%	116.3%	132.6%
**11–20**	3%	21.8%	38.4%	17.8%	45.5%	53.2%
**21–30**	48.3%	57.8%	70.5%	78.7%	53.8%	61.3%
**31–40**	42.5%	49.2%	54.9%	57.4%	56.6%	64.6%
**41–50**	0.5%	0.2%	1.8%	−0.8%	23.2%	27.4%
**51–60**	5.9%	2.3%	0.7%	−5.2%	7.7%	9.1%
**61–70**	21%	19.8%	17.4%	12.2%	12.4%	14%
**71–80**	22.6%	15.1%	15.6%	10.2%	18.9%	21.4%
**81–90**	18.2%	5.6%	6.3%	−1%	14%	16.2%
**91–100**	14.8%	0%	6.1%	−2.8%	6.3%	6.8%

Source: Compiled by the authors. * Data for years 2024 and 2025 are projections.

## Data Availability

The data and R scripts supporting the findings of this study are publicly available in [13], the Zenodo repository at https://doi.org/10.5281/zenodo.17347976 (accessed on 8 January 2026). The repository includes the mortality data used in the analysis, as well as the R scripts required for data processing, modelling, and result generation, allowing full reproducibility of the analysis. The repository includes R scripts that generate simulated outcomes using Monte Carlo methods for uncertainty assessment. No additional empirical data were collected for this study. The original contributions presented in this study are included in the article. Further inquiries can be directed to the corresponding author.
